# Limitations of diabetes pharmacotherapy: results from the Vermont Diabetes Information System study

**DOI:** 10.1186/1471-2296-7-50

**Published:** 2006-08-15

**Authors:** Charles D MacLean, Benjamin Littenberg, Amanda G Kennedy

**Affiliations:** 1 Division of General Internal Medicine, University of Vermont College of Medicine, 371 Pearl Street, Burlington, VT 05401, USA

## Abstract

**Background:**

There are a wide variety of medications available for the treatment of hyperglycemia in diabetes, including some categories developed in recent years. The goals of this study were to describe the glycemic medication profiles in a cohort of adult patients enrolled in primary care, to compare the regimens with measures of glycemic control, and to describe potential contraindicated regimens.

**Methods:**

One thousand and six subjects with diabetes cared for in community practices in the Northeast were interviewed at home at the time of enrollment in a trial of a diabetes decision support system. Laboratory data were obtained directly from the clinical laboratory. Current medications were obtained by direct observation of medication containers by a research assistant.

**Results:**

The median age of subjects was 63 years; 54% were female. The mean A1C was 7.1%, with 60% of subjects in excellent glycemic control (A1C < 7%). Ninety percent of patients were taking 2 or fewer medications for glycemic control, with a range of 0 to 4 medications. Insulin was used by 18%. As the number of diabetes medications increased from 0 to 4, the A1C increased from 6.5% to 9.2% (p < 0.001). The association between glycemic control and number of glycemic medications was confirmed using logistic regression, controlling for potential confounders. Almost 20% of subjects on metformin or thiazolidenediones had potential contraindications to these medications.

**Conclusion:**

Patients with diabetes cared for in primary care are on a wide variety of medication combinations for glycemic control, though most are on two or fewer medications. A greater number of diabetes medications is associated with poorer glycemic control, reflecting the limitations of current pharmacotherapy. One quarter of patients are on glycemic medications with potential contraindications.

## Background

Despite evidence that optimal diabetes care can result in reduced complications and improved economic outcomes, such care is often not achieved [1-4]. Through the 1990s, the number of primary care visits among patients with diabetes listing at least 5 prescription medications increased from 18% to 30%, and the proportion of visits in which more that one medication for glycemic control was listed increased from < 1% to 17%[5]. Oral hypoglycemic agent use increased from 45% to 53%, and combination therapy with insulin and an oral agent increased from 3% to 11% [6].

While there are a wide variety of options for pharmacotherapy of diabetes, there is no one recommended regimen [7]. New classes of medications have been introduced since 1999, including the thiazolidenediones (TZDs), acarbose, and both ultra-short acting and 24-hour insulin analogues. It is not clear how the addition of these new medication classes has changed the landscape of pharmacotherapy of diabetes.

Our goals in this study were: 1) to describe the glycemic medication profiles and the associated level of glycemic control in a cohort of adult patients in primary care settings, and 2) to analyze the medication profiles for potential medication contraindications.

## Methods

This study was part of a larger project, the Vermont Diabetes Information System (VDIS), a cluster-randomized trial of a laboratory-based diabetes decision support system in a sample of 7338 adults with diabetes in northern New England [8]. A detailed description of the recruitment strategy has been reported [8]. In brief, the patient subjects comprised the entire roster of patients with a diagnosis of diabetes (confirmed by the primary care provider) cared for in 64 Primary Care practices in Vermont and adjacent New Hampshire and northern New York. Patients receiving most of their diabetes care from an endocrinologist, or those with significant cognitive impairment were excluded. A field survey was completed at study baseline in a sub-sample of subjects in order to provide a better understanding of the non-laboratory features of diabetes. Patient names were randomly sorted and patients contacted by telephone until a sample of approximately 15% of patients from each practice agreed to an interview. We attempted to contact 4209 patients and reached 1576. Of these, 1006 agreed to be interviewed; they comprise the dataset for our analyses. We have limited data on the non-interviewed patients (N = 6331). They were younger (63 vs. 65 years, p < 0.001 by ttest), slightly less likely to be women (50 vs. 54%, p = 0.01 by chi square analysis), but similar in mean A1C (7.1 vs. 7.1, p = 0.14, ttest).

Demographic information including age, sex, race, ethnicity, education, income, marital status, functional status, and history of comorbid conditions were obtained by questionnaire. Prior to the interview, patients were instructed to gather all current medications, including over the counter preparations, for review by the research assistant. The medication list was ascertained by direct observation of the pill containers with recording of medication name, dose, frequency and route of administration. Adherence was measured by the difference between the number of expected doses of medication per week and the number reported in the past week. Medications taken "as needed" were not included in the adherence measure.

The interviews occurred between July 2003 and March 2005. Most laboratory data were obtained from the patients' local clinical laboratories, which all use the same Diabetes Control and Complications Trial/Epidemiology of Diabetes Interventions and Complications high performance liquid chromatography (HPLC) method for the determination of glycosolated hemoglobin A1C (A1C). Less than 1% of A1C tests were done using the Bayer DCA 2000 immunoassay point of care instrument, which has been shown to compare favorably with the HPLC method [9]. We have not determined the analytic variation in the measurement of A1C between the eleven participating laboratories, but they are all hospital-based, accredited laboratories. The research protocol was carried out in compliance with the Helsinki Declaration and was approved by the Human Subjects Committee of the University of Vermont. The interview subjects provided written informed consent and were reimbursed $20 for their time.

### Statistical approach

We performed a cross sectional analysis of the interviewed subjects at the time of enrollment in the VDIS trial. We used descriptive statistics to describe the proportion of subjects using the various medication regimens, and the subjects with potential contraindications. We used Spearman rank-order correlation [10] and a non-parametric test [11] to assess statistical significance of associations.

To further explore the relationship between glycemic control and the number of medications, we performed logistic regression using A1C < 7% as the outcome variable and the number of glycemic medications as the primary predictor variable [12]. We then adjusted for possible confounding variables that represent social and clinical factors that, if distributed differently among patients on differing numbers of glycemic medications, could confound the relationship between medication count and glycemic control. The potential confounders tested were age, sex, race (white *vs*. other), marital status (married or living as married *vs*. other), high school education (yes/no), income (in seven ordered categories), five insurance types (private, Medicare, Medicaid, Military or Veterans Affairs, none), duration of diabetes, use of insulin, comorbidity (Self-Administered Comorbidity Questionnaire) [13], driving distance to the primary care physician's office [14], functional status (SF-12 Mental and Physical Component Scores) [15], insurance coverage for medications (none, partial, full), visit in the past year with an endocrinologist (yes/no), and visit frequency (number of visits with primary provider in the past month). Further description of the study protocol and variables has been previously reported [8].

All analyses were carried out using Stata version 8.2 (Stata Corporation, College Station, TX).

## Results

The demographic characteristics and level of physiologic control are noted in Table 1. The mean A1C was 7.1% and the proportion of subjects with excellent glycemic control (A1C below 7%) was 60%. Over 95% of the patients were white, with three fourths high school graduates, and 57% earning < $30,000 per year. Mental and physical functioning as measured by the SF-12 Mental Component Summary and Physical Component Summary scales were similar to national norms for patients with diabetes [15].

**Table 1:** Baseline characteristics of the VDIS interview population

Characteristic	N = 1006
Age in years, median (range)	65 (22–93)
Sex, % female	54%
Race (% White)	97%
Education (high school graduate), %	75%
Smoking, %	17%
Income (< $30,000/y), %	59%
Insurance (subjects may have dual coverage)	
Private	58%
Medicare	60%
Medicaid	21%
Military	2%
None	5%
Medication covered by insurance	
None	12%
Partial	72%
Full	16%
Duration of DM, years, median (range)	6.7 (0.2–62)
BMI	
Normal (BMI < 25)	10%
Overweight (BMI 25–29.9)	23%
Obese (BMI >= 30)	67%
A1C %, mean (SD)	7.1 (1.3)
A1C in excellent control [<7%], %	57%
Excellent BP control (<= 130/80), %	25%
Poor BP control (>140/90), %	51%
SF-12 PCS, mean (SD)	41 (12)
SF-12 MCS, mean (SD)	50 (11)
Total prescription medications, median(range)	6 (0–24)

Only 2.1% of the subjects were without health insurance. This reflects the recruitment strategy in which patients under the care of a primary care physician were eligible for participation. Furthermore, Vermont has a relatively low proportion of uninsured persons at 9.9% [16] and a relatively high proportion with Medicaid coverage (17.8%) [17]. The majority of subjects had partial coverage of their medications by insurance, and 14% had full coverage.

The median number of daily prescription medications for all conditions was 6 (range 0–24). Patients reported 90% adherence with the expected number of doses in the week prior to the interview. The diabetes medication profiles of the subjects are shown in Table 2; one quarter were taking no medications, 65% were on one or two diabetes medications, and about 10% were on three or four medications for diabetes. As the number of diabetes medications increases, the proportion of patients with excellent glycemic control (A1C < 7%) decreases steeply and significantly (p < 0.001). The mean A1C increased from 6.5% for patients on no medications to 9.2% for subjects on 4 medications (Spearman rho = 0.35, p < 0.001).

**Table 2:** Medication profiles of the VDIS subjects (N = 1006)

Medication Regimen	N	Proportion of population, %	Excellent control (A1C < 7%), %	Mean A1C, %
Categorized by number of medications				
No diabetes medications	242	25	80	6.5
Monotherapy	402	40	58	7.2
2 Drugs	254	25	46	7.4
3 Drugs	103	10	29	7.6
4 Drugs	5	< 1	0	9.2

Categorized by type of medication				
Any oral medication	670	67	51	7.3
Oral medication(s) alone	578	58	54	7.2
Any insulin therapy	186	19	35	7.8
Insulin alone	94	9	41	7.6
Both oral medication and insulin	92	9	29	7.9

Monotherapy regimen detail				
Sulfonylurea	123	12	58	7.1
Metformin	126	13	66	7.1
TZD	55	5	67	6.8
Insulin	94	9	41	7.6

2-Drug Oral Combination detail				
Sulfonylurea + Metformin	103	10	41	7.5
Sulfonylurea + TZD	49	5	49	7.2
Metformin + TZD	50	5	60	7.1

3 Drug Oral Combination detail				
Sulfonylurea + Metformin + TZD	67	7	35	7.4

Insulin therapy detail				
Insulin glargine	90	9	30	8.0
Any insulin + 1 oral agents	54	5	38	7.8
Any insulin + 2 oral agents	23	2	20	7.9

In the unadjusted logistic regression model, the likelihood of a subject having excellent glycemic control (A1C < 7%) was significantly related to the number of glycemic medications (OR = 0.48/additional medication, *P *< 0.001, 95% CI = 0.42, 0.56). After adjustment for potential confounders (see methods) this relationship did not change appreciably (OR = 0.49/additional medication, *P *< 0.001, 95% CI = 0.41, 0.59).

In an effort to evaluate potentially harmful medication regimens, we looked at the proportion of subjects receiving therapy that may be contraindicated according to the manufacturer's recommendation. These results are shown in Table 3. Metformin is relatively contraindicated in patients with renal dysfunction; we found 10 of 288 subjects on metformin with an abnormal creatinine level (all abnormal values were between 1.5 and 2.0 mg/dl). Congestive heart failure is a relative contraindication to both metformin and thiazolidenediones (TZDs). We found that 14% of subjects on metformin and 18% of subjects on TZD therapy reported a history of heart failure. If all potential contraindications to metformin are combined, 19% of subjects had a potential contraindication. A total of 25% of subjects had a potential contraindication to one of these two medications.

**Table 3:** Potential medication contraindications in VDIS subjects

Potential Contraindication*	Number	% of eligible subjects §
Metformin and Creatinine >= 1.5 mg/dl	15	4%
Metformin and "kidney problems"	15	4%
Metformin and "CHF"	56	14%
Metformin and any of the above	75	19%
TZD and "CHF"	49	19%
Any potential contraindication	252	25%

* Subjects may be in more than one category

§403 subjects on metformin; 264 subjects on TZD

## Discussion

We have described the medication regimens in a cohort of patients with diabetes receiving care in primary care practices in the Northeast. For glycemic control most of these patients (90%) were treated with either no medications, single drug, or two drug combinations. While these patients were treated with what appear to be relatively simple regimens, 40% were not at the American Diabetes Association target for glycemic control of <7%.

There are no clear advantages in terms of blood glucose lowering comparing sulfonylureas, metformin and TZDs. Initial therapy with an oral agent in Type 2 diabetes will decrease the A1C by about 1–2 percentage points [6]. Over time, most patients do not maintain glycemic control on a single agent [5], and combining a second or third oral agent typically produces additive, not synergistic effects. We have demonstrated that as the number of glycemic medications increases, the level of glycemic control worsens. This association persists after controlling for demographic and treatment variables. It most likely reflects confounding by intention: subjects with the highest blood sugar or A1C levels are preferentially put on more aggressive regimens, but these regimens are only partially effective in controlling hyperglycemia.

Two recent studies have addressed the complexity of DM regimens using data from large national datasets. Koro and colleagues, comparing National Health and Nutrition Examination Survey data from 1988 to 2000, showed oral hypoglycemic agent use increased from 45% to 53%, insulin use decreased from 24% to 16% and combination therapy with insulin and an oral agent increased from 3% to 11% [6]. Grant *et al*. using data from the Ambulatory Medical Care Survey from 1991–2000 found that oral hypoglycemic agent use increased from 37% to 51% of visits and insulin use decreased from 25% to 15%[5]. We found an even higher proportion of subjects on oral hypoglycemic agents (67%), and a similar proportion on insulin (18%). The important distinctions between our study and previous reports are: 1) our data collection reflects care in 2003–2005 including therapies introduced since 2000, and 2) our subjects are representative of patients who are receiving care from a primary care provider. With the addition of the TZDs and 24-hour insulin analogues, it would appear that the use of oral medications has been continuing to increase, while insulin use is stable.

In Type 2 DM, insulin is often used after other treatments have failed. Some authors, recognizing that multi-drug oral combinations are not particularly effective, have suggested that insulin is underused [18-20]. While there are barriers to starting insulin in Type 2 DM [21], there is increasing recognition of ways to address those barriers [22]. Because of the complexity of medication options in Type 2 DM and the heterogeneity of the disease process, treatment options should be individualized based on the needs and preferences of the prescriber and the patient.

We identified potential medication contraindications by comparing the medication profiles with self-reported comorbid conditions and with abnormal lab values. Both metformin and the TZDs have CHF as a relative contraindication [23-25]. We found that about 15% of subjects with self-reported CHF were on one or the other of these medications. This finding may indicate a prescribing problem, but is limited by the lack of data regarding the intentions of the prescriber and the inherent inaccuracies of self-report of comorbid conditions. Furthermore, new uncertainty regarding the contraindication to metformin use in CHF was recently introduced by findings that mortality and hospitalization were actually lower among CHF patients in Saskatchewan prescribed metformin versus sulfonylureas [26].

We looked at potential renal contraindications in subjects on metformin therapy and found that fewer that 5% of subjects had either mildly elevated creatinine values (all < 2.0 mg/dl) or self-reported kidney problems. The larger decision support system study in which these subjects are enrolled automatically sends alerts to the physician warning against the use of metformin whenever an abnormal creatinine value is reported, providing a safeguard against continued use. This safeguard was not yet in place when the data were collected.

This study has several strengths. The VDIS study population (n = 7338) comprises the entire roster of patients with diabetes receiving care in the practices enrolled in the larger controlled trial. The interviewed subjects are a randomly selected subset of this population, and are therefore likely to be representative of patients cared for in community primary care settings in the Northeast. Ascertainment of the medication profiles was by direct observation by a research assistant in the patient's home and not subject to the biases inherent in administrative pharmacy databases.

This study also has several limitations. We do not have data on the prescribing intent of the PCPs or side effects experienced by the patients, so we cannot know what barriers may exist to the advancement of pharmacotherapy in an individual patient. We did not include patients whose primary diabetes care is by an endocrinologist, so our results may not generalize to patients cared for in a specialty setting. This may especially exclude patients with highly refractory DM or those using insulin pump therapy. We do not distinguish between patients with Type 1 and Type 2 DM, though from a clinician's point of view, this distinction may be less important than understanding the pathophysiology and response to treatment in an individual patient in order to direct therapy [27,28].

## Conclusion

Patients with diabetes cared for in primary care are on a wide variety of medication combinations for glycemic control, though most are on two or fewer medications. A greater number of diabetes medications is associated with poorer glycemic control, reflecting the limitations of current pharmacotherapy. One quarter of patients are on glycemic medications with potential contraindications.

## Competing interests

The author(s) declare that they have no competing interests.

## Authors' contributions

CDM and BL obtained funding. All authors conceived the research question and study design. CDM performed the data analysis. All authors edited and approved the final manuscript.

## Pre-publication history

The pre-publication history for this paper can be accessed here:



## References

[B1] Harris MI (2000). Health care and health status and outcomes for patients with type 2 diabetes. Diabetes Care.

[B2] Saaddine JB, Engelgau MM, Beckles GL, Gregg EW, Thompson TJ, Narayan KM (2002). A diabetes report card for the United States: quality of care in the 1990s. Ann Intern Med.

[B3] Beckles GL, Engelgau MM, Narayan KM, Herman WH, Aubert RE, Williamson DF (1998). Population-based assessment of the level of care among adults with diabetes in the U.S. Diabetes Care.

[B4] Saydah SH, Fradkin J, Cowie CC (2004). Poor control of risk factors for vascular disease among adults with previously diagnosed diabetes. Jama.

[B5] Grant RW, Pirraglia PA, Meigs JB, Singer DE (2004). Trends in complexity of diabetes care in the United States from 1991 to 2000. Arch Intern Med.

[B6] Koro CE, Bowlin SJ, Bourgeois N, Fedder DO (2004). Glycemic control from 1988 to 2000 among U.S. adults diagnosed with type 2 diabetes: a preliminary report. Diabetes Care.

[B7] ADA (2006). Standards of Medical Care in Diabetes--2006. Diabetes Care.

[B8] MacLean CD, Littenberg B, Gagnon MS, Reardon M, Turner PD, Jordan C (2004). The Vermont Diabetes Information System (VDIS): Study Design and Subject Recruitment for a Cluster Randomized Trial of a Decision Support System in a Regional Sample of Primary Care Practices. Clinical Trials.

[B9] Tamborlane WV, Kollman C, Steffes MW, Ruedy KJ, Dongyuan X, Beck RW, Chase P, Fox LA, Wilson DM, Tsalikian E (2005). Comparison of fingerstick hemoglobin A1c levels assayed by DCA 2000 with the DCCT/EDIC central laboratory assay: results of a Diabetes Research in Children Network (DirecNet) Study. Pediatr Diabetes.

[B10] Spearman C (1904). The proof and measurement of association between two things.. Am J Psychol.

[B11] Cuzick J (1985). A Wilcoxon-type test for trend. Stat Med.

[B12] Hosmer DW, Lemeshow S (2000). Applied Logistic Regression (2nd ed).

[B13] Sangha O, Stucki G, Liang MH, Fossel AH, Katz JN (2003). The Self-Administered Comorbidity Questionnaire: a new method to assess comorbidity for clinical and health services research. Arthritis Rheum.

[B14] Strauss K, MacLean C, Troy A, Littenberg B (2006). Driving distance as a barrier to glycemic control in diabetes. J Gen Intern Med.

[B15] Ware JE, Kosinski M, Turner-Bowker DM, Gandek B (2002). How to Score Version 2 of the SF-12 Health Survey..

[B16] US_Census Income, Poverty, and Health Insurance Coverage in the United States. http://www.census.gov/prod/2004pubs/p60-226.pdf.

[B17] US_Census Health Insurance Coverage Status and Type of Coverage by State and Age for All People. http://pubdb3.census.gov/macro/032005/health/h05_000.htm.

[B18] Riddle MC (2002). The underuse of insulin therapy in North America. Diabetes Metab Res Rev.

[B19] Wright A, Burden AC, Paisey RB, Cull CA, Holman RR (2002). Sulfonylurea inadequacy: efficacy of addition of insulin over 6 years in patients with type 2 diabetes in the U.K. Prospective Diabetes Study (UKPDS 57). Diabetes Care.

[B20] Yki-Jarvinen H (2002). Combination therapy with insulin and oral agents: optimizing glycemic control in patients with type 2 diabetes mellitus. Diabetes Metab Res Rev.

[B21] Goel A, MacLean CD, Walrath D, Rubin A, Huston D, Jones MC, Niquette T, Kennedy AG, Beardall RW, Littenberg B (2004). Adapting root cause analysis to chronic medical conditions. Jt Comm J Qual Saf.

[B22] Polonsky WH, Fisher L, Guzman S, Villa-Caballero L, Edelman SV (2005). Psychological Insulin Resistance in Patients With Type 2 Diabetes: The scope of the problem. Diabetes Care.

[B23] (2004). Glucophage [package insert]. Princeton, NJ: Bristol-Myers Squibb Company..

[B24] (2004). Actos [package insert]. Lincolnshire, IL: Takeda Pharmaceuticals America, Inc; Indianapolis, IN: Eli Lilly and Company..

[B25] (2005). Avandia [package insert]. Research Triangle Park, NC: GlaxoSmithKline..

[B26] Eurich DT, Majumdar SR, McAlister FA, Tsuyuki RT, Johnson JA (2005). Improved clinical outcomes associated with metformin in patients with diabetes and heart failure. Diabetes Care.

[B27] ADA (2005). Diagnosis and Classification of Diabetes Mellitus. Diabetes Care.

[B28] Aye T, Levitsky LL (2003). Type 2 diabetes: an epidemic disease in childhood. Curr Opin Pediatr.

